# Perfect Density Models Cannot Guarantee Anomaly Detection

**DOI:** 10.3390/e23121690

**Published:** 2021-12-16

**Authors:** Charline Le Lan, Laurent Dinh

**Affiliations:** 1 Department of Statistics, University of Oxford, Oxford OX1 3LB, UK; 2 Google Research, Montreal, QC H3B 2Y5, Canada; laurentdinh@google.com

**Keywords:** deep generative modeling, probabilistic modeling, anomaly detection

## Abstract

Thanks to the tractability of their likelihood, several deep generative models show promise for seemingly straightforward but important applications like anomaly detection, uncertainty estimation, and active learning. However, the likelihood values empirically attributed to anomalies conflict with the expectations these proposed applications suggest. In this paper, we take a closer look at the behavior of distribution densities through the lens of reparametrization and show that these quantities carry less meaningful information than previously thought, beyond estimation issues or the curse of dimensionality. We conclude that the use of these likelihoods for anomaly detection relies on strong and implicit hypotheses, and highlight the necessity of explicitly formulating these assumptions for reliable anomaly detection.

## 1. Introduction

Several machine learning methods aim at extrapolating a behavior observed on training data in order to produce predictions on new observations. However, every so often, such extrapolation can result in wrong outputs, especially on points that we would consider infrequent with respect to the training distribution. Faced with unusual situations, whether adversarial [1,2] or just rare [3], a desirable behavior from a machine learning system would be to flag these *outliers* so that the user can assess if the result is reliable and gather more information if it should be necessary [4,5]. This can be critical for applications like medical decision making [6] or autonomous vehicle navigation [7], where such outliers are ubiquitous.

What are the situations that are deemed unusual? Defining these *anomalies* [8,9,10,11,12] manually can be laborious if not impossible, and so generally applicable, automated methods are preferable. In that regard, the framework of *probabilistic reasoning* has been an appealing formalism because a natural candidate for outliers are situations that are *improbable*. Since the true *probability distribution density* $p_{X}^{*}$ of the data is mostly not provided, one would instead use an estimator $p_{X}^{\left(\theta \right)}$ from this data to assess the regularity of a point.

Density estimation has been a particularly challenging task on high-dimensional problems. However, recent advances in *deep probabilistic models*, including variational auto-encoders [13,14,15], deep autoregressive models [16,17,18], and flow-based generative models [19,20,21,22,23,24], have shown promise for density estimation, which has the potential to enable accurate *density-based methods* [25] for anomaly detection.

Yet, several works have observed that a significant gap persists between the potential of density-based anomaly detection and empirical results. For instance [26,27,28] noticed that generative models trained on a benchmark dataset (e.g., CIFAR-10, [29]) and tested on another (e.g., SVHN, [30]) are not able to identify the latter as an outlier with current methods. Different hypotheses have been formulated to explain that discrepancy, ranging from the *curse of dimensionality* [31] to a significant mismatch between $p_{X}^{\left(\theta \right)}$ and $p_{X}^{*}$ [26,32,33,34,35,36].

**Figure 1:**
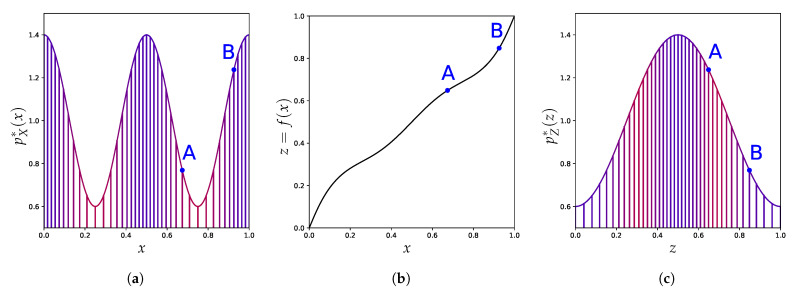
An invertible change of representation can affect the relative density between two points A and B, which has been interpreted as their relative regularity. (**a**) An example of a distribution density $p_{X}^{*}$. (**b**) Example of an invertible function *f* from [0,1] to [0,1]. (**c**) Resulting density $p_{Z}^{*}$ as a function of the new axis z=f(x). In (**a**,**c**) points with high original density $p_{X}^{*}\left(x\right)$ are in blue and red for low original density.

In this work, we propose a new perspective on this discrepancy and challenge the expectation that density estimation should always enable anomaly detection. We show that the aforementioned discrepancy persists even with perfect density models, and therefore goes beyond issues of estimation, approximation, or optimization errors [37]. We highlight that this issue is pervasive as it occurs even in low-dimensional settings and for a variety of density-based methods for anomaly detection. Focusing on the continuous input case, we make the following contributions:

- Similar to classification, we propose in Section 3 a principle of invariance to formalize the underlying assumptions behind the current practice of (deep) density-based methods.
- We use the well-known change of variables formula for probability density to show in Section 4 how these density-based methods are not invariant to reparametrization (see Figure 1) and contradict this principle. We demonstrate the extent of the issues with current practices by building adversarial cases, even under strong distributional constraints.
- Given the resulting tension between the use of these anomaly detection methods and their lack of invariance, we focus in Section 5 on the importance of explicitly incorporating prior knowledge into (density-based) anomaly detection methods as a more promising avenue to reconcile this tension.

## 2. Density-Based Anomaly Detection

In this section, we present existing density-based anomaly detection approaches that are central to our analysis. Seen as methods without explicit prior knowledge, they aim at unambiguously defining outliers and inliers.

### 2.1. Unsupervised Anomaly Detection: Problem Statement

**Figure 2:**
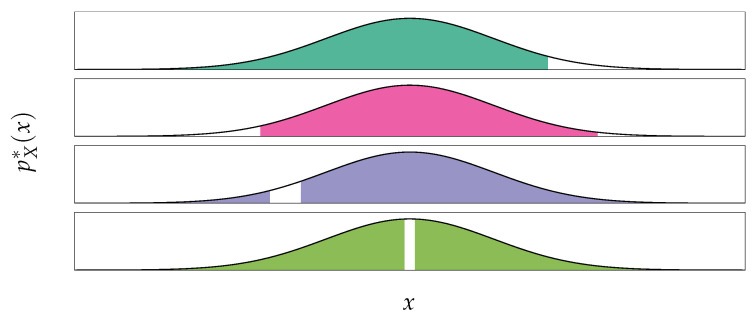
There is an infinite number of ways to partition a distribution in two subsets, $X_{in}$ and $X_{out}$ such that $P_{X}^{*}\left(X_{in}\right)=0.95$. Here, we show several choices for a standard Gaussian $p_{X}^{*}=N\left(0,1\right)$.

Unsupervised anomaly detection is a classification problem [38,39,40], where one aims at distinguishing between regular points (*inliers*) and irregular points (*outliers*). However, as opposed to the usual classification task, labels distinguishing inliers and outliers are not provided for training, if outliers are even provided at all. Given an input space $X\subseteq R^{D}$, the task can be summarized as partitioning this space between the subset of outliers $X_{out}$ and the subset of inliers $X_{in}$, i.e., $X_{out}\cup X_{in}=X$ and $X_{out}\cap X_{in}=⌀$. When the training data is distributed according to the probability measure $P_{X}^{*}$ (with density $p_{X}^{*}$, that we assume in the rest of the paper to be such that $\forall x\in X,p_{X}^{*}\left(x\right)>0$) one would usually pick the set of regular points $X_{in}$ such that this set contains the majority (but not all) of the mass (e.g., 95%) of this distribution [39], i.e., $P_{X}^{*}\left(X_{in}\right)=1-\alpha \in \left(\frac{1}{2},1\right)$. However, for any given α, there exists in theory an infinity of corresponding partitions into $X_{in}$ and $X_{out}$ (see Figure 2). How are these partitions picked to match our intuition of inliers and outliers? In particular, how can we *learn* from data to discriminate between inliers and outliers (without of course predefining them)? We will focus in this paper on recently used methods based on probability density.

### 2.2. Density Scoring Method

When talking about outliers—infrequent observations—the association with probability can be quite intuitive. For instance, one would expect an anomaly to happen rarely and be unlikely. Since the language of statistics often associate the term *likelihood* with quantities like $p_{X}^{\left(\theta \right)}\left(x\right)$, one might consider an unlikely sample to have a low "likelihood", that is, a low probability density $p_{X}^{*}\left(x\right)$. Conversely, regular samples would have a high density $p_{X}^{*}\left(x\right)$ following that reasoning. This is an intuition that is not only prevalent in several modern anomaly detection methods [25,28,34,41,42,43] but also in techniques like low-temperature sampling [44] used for example in Kingma and Dhariwal [21] and parmar et al. [45].

**Figure 3:**
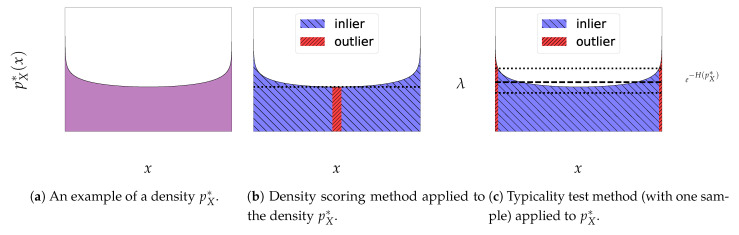
Illustration of different density-based methods applied to a particular one-dimensional distribution $p_{X}^{*}$. Outliers are in red and inliers are in blue. The thresholds are picked so that inliers include 95% of the mass. In (**b**), inliers are considered as the points with density above the threshold λ>0 while in (**c**), they are the points whose log-density are in the ϵ-interval around the negentropy $-H(p_{X}^{*})$.

The associated approach, described in Bishop [25], consists in defining the inliers as the points whose density exceed a certain threshold λ>0 (for example, chosen such that inliers include a predefined amount of mass, e.g., 95%), making the modes the most regular points in this setting. $X_{out}$ and $X_{in}$ are then respectively the lower-level and upper-level sets $\left\{x\in X,p_{X}^{*}\left(x\right)\leq \lambda \right\}$ and $\left\{x\in X,p_{X}^{*}\left(x\right)>\lambda \right\}$ (see Figure 3b).

### 2.3. Typicality Test Method

The *Gaussian Annulus theorem* [46] generalized in [47] attests that most of the mass of a high-dimensional standard Gaussian $N(0,I_{D})$ is located close to the hypersphere of radius $\sqrt{D}$. However, the mode of its density is at the center 0. A natural conclusion is that the *curse of dimensionality* creates a discrepancy between the density upper-level sets and what we expect as inliers [26,31,48,49]. This motivated Nalisnick et al. [31] to propose another method for testing whether a point is an inlier or not, relying on a measure of its *typicality*. This method relies on the notion of *typical set* [50] defined by taking as inliers points whose average log-density is close to the average log-density of the distribution (see Figure 3c).

**Definition** **1** ([50]). Given independent and identically distributed elements ${\left(x^{\left(n\right)}\right)}_{n\leq N}$ from a distribution with density $p_{X}^{*}$, the typical set $A_{\epsilon }^{\left(N\right)}\left(p_{X}^{*}\right)\subset X^{N}$ is made of all sequences that satisfy

$$\begin{array}{c} |H\left(p_{X}^{*}\right)+\frac{1}{N}\sum_{n=1}^{N}\log p_{X}^{*}\left(x^{\left(n\right)}\right)|\leq \epsilon , \end{array}$$

where $H\left(p_{X}^{*}\right)=-E\left[\log p_{X}^{*}\left(X\right)\right]$ is the (differential) entropy and ϵ>0 a constant.

This method matches the intuition behind the Gaussian Annulus theorem on the set of inliers of a high-dimensional standard Gaussian. Indeed, using a concentration inequality, we can show that $\lim_{N\to +\infty }\left(P_{{\left(X_{i}\right)}_{1\leq n\leq N}}^{*}\left(A_{\epsilon }^{\left(N\right)}\right)\right)=1$, which means that with *N* large enough, $A_{\epsilon }^{\left(N\right)}\left(p_{X}^{*}\right)$ will contain most of the mass of ${\left(p_{X}^{*}\right)}^{N}$, justifying the name *typicality*.

## 3. The Role of Reparametrization

Density-based anomaly detection is applied in practice [25,28,34,41,42,43] as follows: first, learn a density estimator $p_{X}^{\left(\theta \right)}$ to approximate the data density $p_{X}^{*}$, and then plug that estimate in the density-based methods from Section 2.2 and Section 2.3 to discriminate between inliers and outliers. Recent empirical failures [3,26,27] of this procedure applied to density scoring have been attributed to the discrepancy between $p_{X}^{\left(\theta \right)}$ and $p_{X}^{*}$ [28,33,34,35,48]. Instead, we choose in this paper to question the fundamental assumption that these density-based methods should result in a correct classification between outliers and inliers.

### 3.1. A Binary Classification Analogy

We start by studying the desired behavior of a classification method under infinite data and capacity, a setting where the user is provided with a perfect density model $p_{X}^{\left(\theta \right)}=p_{X}^{*}$.

In Magritte [51], the author reminds us that the input *x* we use is merely an arbitrary representation of the studied object (in other words, “a map is not the territory” [52]), standardized here to enable the construction of a large-scale homogeneous dataset to train on [53]. This is after all the definition of a random variable x=X(ω), which is by definition a function from the underlying outcome ω to the corresponding observation *x*. For instance, in the case of object classification, ω is the object while X(ω) is the image (produced as a result of lighting, camera position and pose, lenses, and the image sensor). For images, a default representation is the bitmap one. However, this choice of representation remains arbitrary and practitioners have also trained their classifier using pretrained features instead [54,55,56], Jpeg representation [57], encrypted version of the data [58,59], or other resulting transformations $f\left(x\right)=f\left(X(\omega )\right)$, without modifying the associated labels. In particular, if *f* is invertible, $f\left(x\right)=f\left(X(\omega )\right)$ contains the same information about ω as x=X(ω). Therefore both representations should be classified the same, as we associate the label with the underlying outcome ω. If $c^{*}$ is the perfect classifier on *X*, then $c^{*}\circ f^{-1}$ should be the perfect classifier on f(X)=(f∘X) to assess the label of ω, since $\left(c^{*}\circ f^{-1}\right)\left(f(x)\right)=c^{*}\left(x\right)$.

As an illustration, we can consider the transition from of a cartesian coordinate system ${\left(x_{i}\right)}_{i\leq D}$ to a hyperspherical coordinate system, consisting of a radial coordinate r>0 and (D−1) angular coordinates ${\left(\phi_{i}\right)}_{i<D}$,

$$\begin{array}{cc} \forall d<D,\;x_{d} & =r\left(\prod_{i=1}^{d-1}\sin(\phi_{i})\right)\cos\left(\phi_{d}\right)\\ x_{D} & =r\left(\prod_{i=1}^{D-2}\sin(\phi_{i})\right)\sin\left(\phi_{D-1}\right), \end{array}$$

where for all i∈{1,2,…,D−2},$\phi_{i}\in \left[0,\pi \right)$ and $\phi_{D-1}\in \left[0,2\pi \right)$. While significantly different, those two systems of coordinates (or representations) describe the same vector and are connected by an invertible map *f*. In a Cartesian coordinate system, an optimal classifier $c^{*}\left(x\right)=1\left(\sum x_{i}^{2}>1\right)$ would become in a hyperspherical representation $\left(c^{*}\circ f^{-1}\right)\left(r,\phi \right)=1\left(r^{2}>1\right)$.

The ability to learn the correct classification rule from infinite data and capacity, regardless of the representation used (or with minimal requirement), is a fundamental requirement (albeit weak) for a machine learning algorithm, and hence an interest in universal approximation properties, see [60,61,62]. While we do not dismiss the important role of the input representation as an inductive bias (e.g., using pretrained features as inputs), its influence should in principle dissipate entirely in the infinite data and capacity regime and the resulting solution from this ideal setting should be unaffected by this inductive bias. In ideal conditions, solutions to classification should be invariant to any invertible change of representation.

We consider this is in fact one of key tenets behind deep learning [63] and feature engineering/learning in general [64].

### 3.2. A Principle for Anomaly Detection Methods

Current practices of deep anomaly detection commonly include the use of deep density models on either default input feature [26,27,28,31,34,42] or features learned independently from the anomaly detection task [6,48,65,66]. The process of picking a particular input representation is rarely justified in the context of density-based anomaly detection, which suggests that a similar implicit assumption is being used: *the status of inlier/outlier corresponds to the underlying outcome ω behind an input feature x=X(ω), whose only role is to inform us on ω*. As described in Section 2.1, the goal of anomaly detection is, like classification, to discriminate (although generally in an unsupervised way) between inliers and outliers. Similarly to classification, the label of inlier/outlier of an underlying outcome should remain invariant to reparametrization in an infinite data and capacity setting, especially since information about ω (and whether the outcome is anomalous or not) is conserved under an invertible transformation up to numerical instabilities, see [67]. We consider the following principle:

**Principle.**  *In an infinite data and capacity setting, the result of an anomaly detection method should be invariant to any continuous invertible reparametrization f*.

This principle is coherent with the fact that, with *f* invertible, the set of outliers $X_{out}$ remains a low probability subset as $P_{X}\left(X_{out}\right)=P_{f(X)}\left(f(X_{out})\right)$ and $\forall x\in X,\;x\in X_{out}\Leftrightarrow f\left(x\right)\in f\left(X_{out}\right)$. However, density-based methods do not follow this principle as densities are not representation-invariant. In particular, the change of variables formula [68], also used in Dinh et al. [69], Tabak and Turner [19], Rezende and Mohamend [70], formalizes a simple intuition of this behavior: where points are brought closer together the density increases whereas this density decreases when points are spread apart.

**Figure 4:**
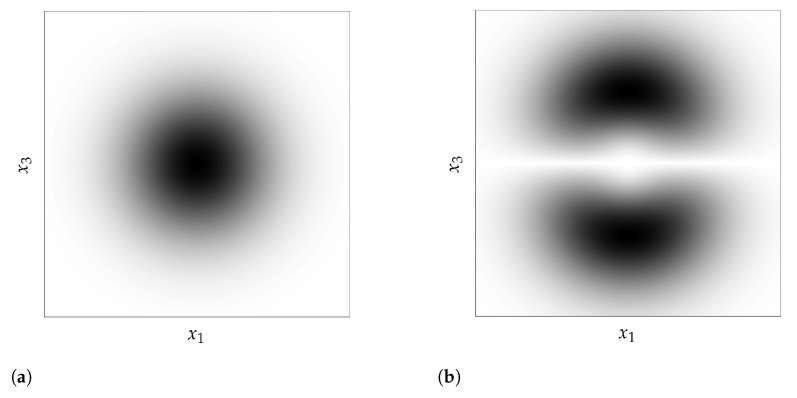
Illustration of the change of variables formula for a three-dimensional standard Gaussian distribution with a change of coordinate system, from Cartesian to hyperspherical (where density follows the intuition of the Gaussian Annulus Theorem) (**a**) A three-dimensional standard Gaussian distribution density in Cartesian coordinates on the hyperplane defined by $x_{2}=0$. (**b**) A three-dimensional standard Gaussian distribution density in hyperspherical coordinates (plotted in Cartesian coordinates) on the hyperplane defined by $x_{2}=0$.

The formula itself is written as:

$$p_{f(X)}^{*}\left(f(x)\right)=p_{X}^{*}\left(x\right){\left|\frac{\partial f}{\partial x^{T}}\left(x\right)\right|}^{-1}$$

where $|\frac{\partial f}{\partial x^{T}}\left(x\right)|$ is the Jacobian determinant of *f* at *x*, a quantity that reflects a local change in volume incurred by *f*. Figure 1 already illustrates how the function *f* Figure 1b can spread apart points close to the extremities to decrease the corresponding density around x=0 and x=1, and, as a result, turns the density on the left Figure 1a into the density on the right Figure 1c. Figure 4 shows how much a simple change of coordinate system, from Cartesian (Figure 4a) to hyperspherical (Figure 4b), can significantly affect the resulting density associated with a point. This comes from the Jacobian determinant of this change of coordinates:

$$r^{D-1}\left(\prod_{d=1}^{D-1}{\left(\sin(\phi_{d})\right)}^{D-d-1}\right).$$

With these examples, one can wonder to which degree an invertible change of representation can affect the density and thus the anomaly detection methods presented in Section 2.2 and Section 2.3 that use it. This is what we explore in Section 4.

## 4. Leveraging the Change of Variables Formula

### 4.1. Uniformization

We start by showing that unambiguously defining outliers and inliers with any density-based approach becomes impossible when considering a particular type of invertible reparametrization of the problem, irrespective of dimensionality.

**Figure 5:**
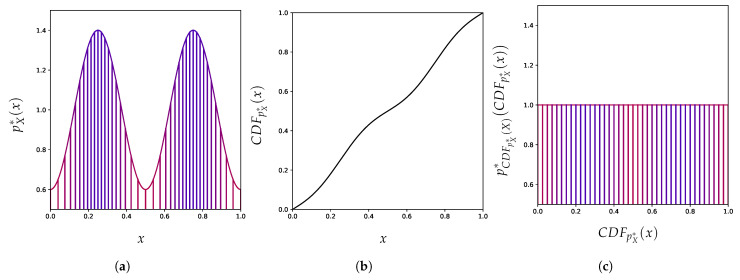
Illustration of the one-dimensional case version of a Knothe-Rosenblatt rearrangement, which is just the application of the cumulative distribution function $CDF_{p_{X}^{*}}$ on the variable *x*. Points *x* with high density $p_{X}^{*}\left(x\right)$ are in blue and points with low density $p_{X}^{*}\left(x\right)$ are in red. (**a**) An example of a distribution density $p_{X}^{*}$. (**b**) The corresponding cumulative distribution function $CDF_{p_{X}^{*}}$. (**c**) The resulting density from applying $CDF_{p_{X}^{*}}$ to $X∼p_{X}^{*}$ is $p_{CDF_{p_{X}^{*}}\left(X\right)}^{*}=U\left(\left[0,1\right]\right)$.

Under weak assumptions, one can map any distribution to a uniform distribution using an invertible transformation [71]. This is in fact a common strategy for sampling from complicated one-dimensional distributions [72]. Figure 5 shows an example of this where a bimodal distribution (Figure 5a) is pushed through an invertible map (Figure 5b) to obtain a uniform distribution (Figure 5c).

To construct this invertible uniformization function, we rely on the notion of Knothe-Rosenblatt rearrangement [73,74]. A Knothe-Rosenblatt rearrangement notably used in [71] is defined for a random variable *X* distributed according to a strictly positive density $p_{X}^{*}$ with a convex support X, as a continuous invertible map $f^{\left(KR\right)}$ from X onto ${\left[0,1\right]}^{D}$ such that $f^{\left(KR\right)}\left(X\right)$ follows a uniform distribution in this hypercube. This rearrangement is constructed as follows: $\forall d\in \left\{1,\ldots ,D\right\},f^{\left(KR\right)}\left(x\right)=CDF_{p_{X_{d}|X_{<d}}^{*}}\left(x_{d}|x_{<d}\right)$ where $CDF_{p}$ is the cumulative distribution function corresponding to the density *p*.

In these new coordinates, neither the density scoring method nor the typicality test approach can discriminate between inliers and outliers in this uniform *D*-dimensional hypercube ${\left[0,1\right]}^{D}$. Since the resulting density $p_{f^{\left(KR\right)}\left(X\right)}^{*}=1$ is constant, the density scoring method attributes the same regularity to every point or set of points. Moreover, a typicality test on $f^{\left(KR\right)}\left(X\right)$ will always succeed as

$$\begin{array}{c} \forall \epsilon >0,N\in N^{*},\forall {\left(x^{\left(n\right)}\right)}_{n\leq N},T_{typ}\left(p_{f^{\left(KR\right)}\left(X\right)}^{*},{\left(x^{\left(n\right)}\right)}_{n\leq N}\right)=0\leq \epsilon . \end{array}$$

However, these uniformly distributed points are merely a different representation of the same initial points. Therefore, if the identity of the outliers is ambiguous in this uniform distribution, then anomaly detection in general should be as difficult.

### 4.2. Arbitrary Scoring

**Figure 6:**
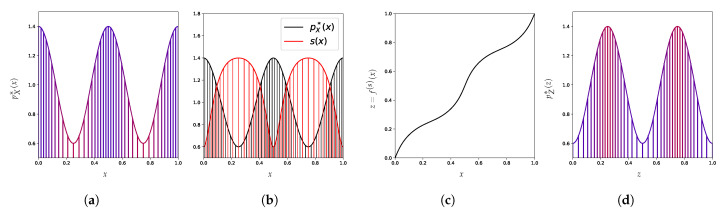
Illustration of how we can modify the space with an invertible function so that each point *x* follows a predefined score. In (**a**,**b**) points with high original density $p_{X}^{*}\left(x\right)$ are in blue and red for low original density. (**a**) An example of a distribution density $p_{X}^{*}$. (**b**) The density $p_{X}^{*}$ (in black) and the desired density scoring *s* (in red). (**c**) A continuous invertible reparametrization $z=f^{\left(s\right)}\left(x\right)$ such that $p_{Z}^{*}\left(z\right)=s\left(x\right)$. (**d**) Resulting density $p_{Z}^{*}$ from applying $f^{\left(s\right)}$ to $X∼p_{X}^{*}$ as a function of $z=f^{\left(s\right)}\left(x\right)$.

We find that it is possible to build a reparametrization of the problem to impose to each point an arbitrary density level in the new representation. To illustrate this idea, consider some points from a distribution whose density is depicted in Figure 6a and a score function indicated in red in Figure 6b. In this example, high-density regions correspond to areas with low score value (and vice-versa), such that the ranking from the densities is reversed with this new score. Given that desired score function, we show how to systematically build a reparametrization (depicted in Figure 6c) such that the density in this new representation (Figure 6d) now matches the desired score, which can be designed to mislead density-based methods into a wrong classification of anomalies by modifying a single dimension (in a potentially high-dimensional input vector).

**Proposition** **1.** For any random variable $X∼p_{X}^{*}$ with $p_{X}^{*}$ strictly positive (with X convex) and any measurable continuous function $s:X\to R_{+}^{*}$ bounded below by a strictly positive number, there exists a continuous bijection $f^{\left(s\right)}$ such that for any $x\in X,p_{f^{\left(s\right)}\left(X\right)}^{*}\left(f^{\left(s\right)}\left(x\right)\right)=s\left(x\right)$.

**Proof.**  We write *x* to denote $\left(x_{1},\cdots ,x_{D-1},x_{D}\right)$ and $\left(x_{<D},t\right)$ for $\left(x_{1},\cdots ,x_{D-1},t\right)$. Let $f^{\left(s\right)}:X\to Z\subset R^{D}$ be a function such that

$${\left(f^{\left(s\right)}\left(x\right)\right)}_{D}=\int_{0}^{x_{D}}\frac{p_{X}^{*}\left((x_{<D},t)\right)}{s\left((x_{<D},t)\right)}dt,$$

and $\forall d\in \left\{1,\ldots ,D-1\right\},{\left(f^{\left(s\right)}\left(x\right)\right)}_{d}=x_{d}$. As *s* is bounded below, $f^{\left(s\right)}$ is well defined and invertible. □

By the change of variables formula,

$$\begin{array}{c} \forall x\in X,\;p_{f^{\left(s\right)}\left(X\right)}^{*}\left(f^{\left(s\right)}\left(x\right)\right)=p_{X}^{*}\left(x\right)\cdot {\left|\frac{\partial f^{\left(s\right)}}{\partial x^{T}}\left(x\right)\right|}^{-1}=p_{X}^{*}\left(x\right)\cdot {\left(\frac{p_{X}^{*}\left(x\right)}{s(x)}\right)}^{-1}=s\left(x\right). \end{array}$$

If $X_{in}$ and $X_{out}$ are respectively the true sets of inliers and outliers, we can pick a ball $A\subset X_{in}$ such that $P_{X}^{*}\left(A\right)=\alpha <0.5$, we can choose *s* such that for any x∈(X\A), s(x)=1 and for any x∈A,s(x)=0.1. With this choice of *s* (or a smooth approximation) and the function $f^{\left(s\right)}$ defined earlier, both the density scoring and the (one-sample) typical set methods will consider the set of inliers to be (X\A) while $X_{out}\subset \left(X\backslash A\right)$, making their results completely wrong. While we can also reparametrize the problem so that these methods may succeed, e.g., a parametrization where anomalies have low density for the density scoring method, such a reparametrization requires knowledge of $\left(p_{X}^{*}/s\right)\left(x\right)$. Without any constraint on the space considered, individual densities can be arbitrarily manipulated, which reveals how little this quantity says about the underlying outcome in general.

### 4.3. Canonical Distribution

Our analysis from Section 4.2 revealing that densities or low typicality regions are not sufficient conditions for an observation to be an anomaly, whatever its distribution or its dimension, we are now interested in investigating whether additional stronger assumptions can lead to some guarantees for anomaly detection. Motivated by several representation learning algorithms which attempt to learn a mapping to a predefined distribution, e.g., a standard Gaussian, see [13,14,19,65,75], we consider the more restricted setting of a fixed distribution of our choice, whose regular regions could for instance be known. Surprisingly, we find that it is possible to exchange the densities of an inlier and an outlier even within a canonical distribution.

**Proposition** **2.** For any strictly positive density function $p_{X}^{*}$ over a convex space $X\subseteq R^{D}$ with D≥2, for any $x_{in},x_{out}$ in the interior $X^{o}$ of X, there exists a continuous bijection f:X→X such that $p_{X}^{*}=p_{f(X)}^{*}$, $p_{f(X)}^{*}\left(f\left(x^{\left(in\right)}\right)\right)=p_{X}^{*}\left(x^{\left(out\right)}\right)$, and $p_{f(X)}^{*}\left(f\left(x^{\left(out\right)}\right)\right)=p_{X}^{*}\left(x^{\left(in\right)}\right)$.

**Figure 7:**
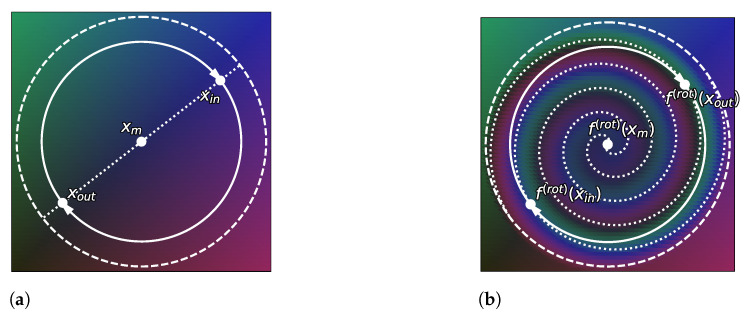
Illustration of the norm-dependent rotation, a locally-acting bijection that allows us to swap two different points while preserving a uniform distribution (as a volume-preserving function). (**a**) Points $x_{in}$ and $x_{out}$ in a uniformly distributed subset. $f^{\left(rot\right)}$ will pick a two-dimensional plane and use the polar coordinate using the mean $x_{m}$ of $x_{in}$ and $x_{out}$ as the center. (**b**) Applying a bijection $f^{\left(rot\right)}$ exchanging the points $x_{in}$ and $x_{out}$. $f^{\left(rot\right)}$ is a rotation depending on the distance from the mean $x_{m}$ of $x_{in}$ and $x_{out}$ in the previously selected two-dimensional plane.

**Figure 8:**
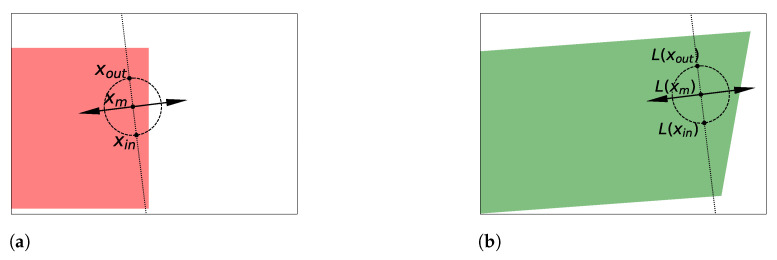
We illustrate how, given $x_{in}$ and $x_{out}$ in a uniformly distributed hypercube ${\left[0,1\right]}^{D}$, one can modify the space such that $f^{\left(rot\right)}$ shown in Figure 7 can be applied without modifying the distribution. (**a**) When taking two points $x_{in}$ and $x_{out}$ inside the hypercube ${\left[0,1\right]}^{D}$, there is sometimes no $L_{2}$-ball centered in their mean $x_{m}$ containing both $x_{in}$ and $x_{out}$. (**b**) However, given $x_{in}$ and $x_{out}$, one can apply an invertible linear transformation *L* such that there exists a $L_{2}$-ball centered in their new mean $L(x_{m})$ containing both $L(x_{in})$ and $L(x_{out})$. If the distribution was uniform inside ${\left[0,1\right]}^{D}$, then it is now also uniform inside $L\left({\left[0,1\right]}^{D}\right)$.

**Proof.**  The proof is given in Appendix A. It relies on the transformation depicted in Figure 7, which can swap two points while acting in a very local area. If the distribution of points is uniform inside this local area, then this distribution will be unaffected by this transformation. To come to this, we use the uniformization method presented in [71], along with a linear function to fit this local area inside the support of the distribution (see Figure 8). Once those two points have been swapped, we can reverse the functions preceding this swap to recover the original distribution overall. □

**Figure 9:**
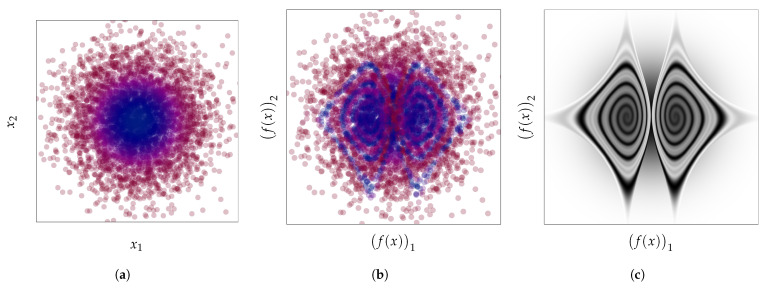
Application of the bijection from Figure 7 to a standard Gaussian distribution $N(0,I_{2})$ leaving it an overall invariant. (**a**) Points sampled from $p_{X}^{*}=N\left(0,I_{2}\right)$. (**b**) Applying a bijection *f* that preserves the distribution $p_{f(X)}^{*}=N\left(0,I_{2}\right)$ to the points in Figure 9a. (**c**) The original distribution $p_{X}^{*}$ with respect to the new coordinates f(x): $p_{X}^{*}\circ f^{-1}$.

Since the resulting distribution $p_{f(X)}^{*}$ is identical to the original distribution $p_{X}^{*}$, their entropies are the same $H\left(p_{f(X)}^{*}\right)=H\left(p_{X}^{*}\right)$. Hence, when $x_{in}$ and $x_{out}$ are respectively an inlier and an outlier, whether in terms of density scoring or typicality, there exists a reparametrization of the problem conserving the overall distribution while still exchanging their status of inlier/outlier. We provide an example applied to a standard Gaussian distribution in Figure 9.

This result is important from a representation learning perspective and a complement to the general non-identifiability result in several representation learning approaches [71,76]. It means that learning a representation with a predefined, well-known distribution and knowing the true density $p_{X}^{*}$ are not sufficient conditions to control the individual density of each point and accurately distinguish outliers from inliers.

### 4.4. Practical Consequences for Anomaly Detection

We showed that the choice of representation can heavily influence the output of the anomaly detection methods described in Section 2.2 and Section 2.3.

#### 4.4.1. Learning a Representation by Applying Explicit Transformations f

Surprisingly, this problem can persist even when the learned representation is lower-dimensional, contains *only* the relevant information for the task, and is axis-aligned with semantic variables, since a reasoning similar to Section 4.2 can be applied using axis-aligned bijections to tamper with densities. If a recent review [12] has highlighted the importance of the choice of representation in the context of low-level/high-level anomalies, our result goes further and shows that a problem still persists as even high-level information can be invertibly reparametrized to impose an arbitrary density-based ranking. This leads us to believe that characterizing which representations are suitable for density-based methods (to conform with human expectations) cannot be answered in the absence of prior knowledge (see Section 4.2), e.g., on the distribution of anomalies.

#### 4.4.2. Arbitrary Input Representation Result from Implicit Transformations f

While (to our knowledge) input features are rarely designed or heavily tampered with to obfuscate density-based methods in practice, input features can often be the result of a system not fully understood end-to-end, that is of some *implicit transformations f*, as to how they influence the task of anomaly detection. For instance, cameras used can be tuned to different tasks and the spectral response of film and image sensors has been tuned to maximize performance on the “Shirley Card” [77,78]. Images can also go through processing techniques like high-dynamic range imaging [79] or arbitrary downsampling as in [29,30,80].

**Figure 10:**
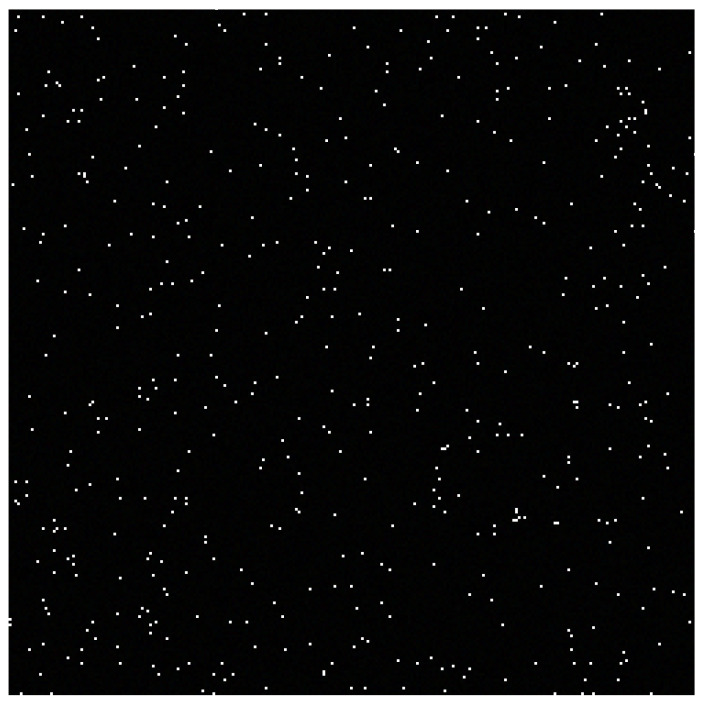
We generated $5^{6}$ pixels according to the procedure described and concatenated them in a single 125×125 RGB bitmap image for an easier visualization. While, visual intuition would suggest that white pixels are the outliers in this figure, density-based definitions of anomalies described Section 2.2 (density scoring) and Section 2.3 (typicality) would consider a specific dark shade of gray to be the outlier.

It is well-understood in *representation learning* [64] that the default input features handed to the learning algorithm are rarely well-tuned to the task it tries to solve, e.g., euclidean distance rarely follows a notion of semantic distance, see [81]. Figure 10 provides an example where these methods fail in pixel space despite being endowed with a perfect density model. Details about its construction and analysis are provided below.

We generate $5^{6}$ individual pixels as three-dimensional vectors according to a distribution built as follows: let $p_{w}=U\left({\left[255,256\right]}^{3}\right)$ (corresponding to the color white), $p_{b}=U\left({\left[0,10\right]}^{3}\right)$ (corresponding to shades of black), and $p_{out}=U\left({\left[10,11\right]}^{3}\right)$ (corresponding to a dark shade of grey) be distributions with disjoint supports. We consider pixels following the distribution

$$\begin{array}{cc} p_{X}\left(x\right)= & \beta \cdot p_{out}\left(x\right)\\ +(1- & \beta )\left(\alpha \cdot p_{w}\left(x\right)+\left(1-\alpha \right)\cdot p_{b}\left(x\right)\right), \end{array}$$

where $\alpha =1001^{-3}$ and $\beta =10^{-4}$. Once generated, we concatenate these pixels in a 125×125 RGB bitmap image in Figure 10 for a more convenient visualization.

Visually, a common intuition would be to consider white pixels to be the anomalies in this figure. However, following a construction similar to Section 4.2, the final densities corresponding to pixels from $p_{w}$ (equal to α(1−β)) and $p_{b}$ (equal to $\left(1-\alpha \right)\left(1-\beta \right)10^{-3}$) are equal to $1001^{-3}\left(1-10^{-4}\right)\approx 10^{-3}$, and the final density corresponding to pixels from $p_{out}$ (equal to β) is $10^{-4}$. Therefore, none of the methods presented in Section 2.2 (density scoring) and Section 2.3 (one-sample typicality) would consider the white pixels (in ${\left[255,256\right]}^{3}$) as outliers. They would only classify the pixels of a particular dark shade of gray in ${\left[10,11\right]}^{3}$ as outliers.

Given the considerable influence, the choice of input representation has on the output of even the true data density function $p_{X}^{*}$, one should question the strong but understated assumption behind current practices that (density-based) anomaly detection methods applied on *default input representations* decontextualized from their design process [82], *representations orthogonally learned from the task*, or *even obtained by filtering noise variables* (non-semantic) ought to result in proper outlier classification.

## 5. Promising Avenues for Unsupervised Density-Based Anomaly Detection

While anomaly detection can be an ill-posed problem as mentioned in [26,31,48] without *prior knowledge*, several approaches are more promising by making this prior knowledge more explicit. We highlighted the strong dependence of density-based anomaly detection methods on a choice of representation, which needs to be justified as it is crucial to the success of the approach. This was proven by using the change of variables formula, which describes how the density function varies with respect to a reparametrization. If we consider the fundamental definition of a density as a Radon-Nikodym derivative $p_{X}^{*}=\frac{dP_{X}^{*}}{d\mu_{X}}$ with respect to a base measure (here the Lebesgue measure $\mu_{X}$ in X), we notice that this variation stems from a change of “denominator”: the Lebesgue measure corresponding to X is different to the one corresponding to another space Z (the Jacobian determinant accounting for this mismatch $\mu_{X}\circ X\neq \mu_{Z}\circ Z$).

A way to incorporate more transparently the choice of representation is to consider a similar fraction. For example, *density ratio methods* [83] score points using a ratio $p_{X}^{*}/p_{BG}$ between two densities. The task is then to figure out whether a point comes from a regular source (the foreground distribution in the numerator) or an anomalous source (the background distribution in the denominator). The concurrent work [84] also draws a similar conclusion showing that no test can distinguish between a given source distribution and an unspecified outlier distribution better than random chance. In Bishop [25], the density scoring method has been interpreted as a density ratio method with a default uniform density function. More refined methods can be used as a background distribution, e.g., $p_{X}^{*}$ convolved with a noise distribution [85], the implicit distribution of a compressor [86], or a mixture including $p_{X}^{*}$ as a component, i.e., a “superset”, see [87]. In addition to being more transparent with respect to its underlying assumptions, density ratio methods are invariant to invertible reparametrization.

While appealing in their property, density ratio methods still require the explicit definition of a background distribution, an explicit guess on how the anomalies should be distributed. It is actually possible in some cases to be more intentional in the definition of this denominator. For example, for exploration in reinforcement learning, Houthooft et al. [88] and Bellemare et al. [89] use an (invertible) reparametrization-invariant proxy for potential information gain.

## 6. Discussion and Limitations

We discussed the ill-defined (and arguably subjective) notion of *outlier* or *anomaly*, which several works attempted to characterize through a seemingly clearer notion of probability density used in the density scoring and typicality test methods. We show in this paper that an undesirable degree of freedom persists in how density functions can be manipulated by an arbitrary choice of representation, rarely set to fit the task. We consider that the lack of attention paid to this crucial element has undermined the foundations of these off-the-shelf methods, potentially providing a simpler explanation to their empirical failures studied in [26,27,28,32,33,34] as a discrepancy with unstated prior assumptions.

We conclude that being more intentional about integrating prior knowledge explicitly in density-based anomaly detection algorithms then becomes essential to their success.

Although a similar issue persists in practice for *discrete spaces* as noted in [49], where outputs with highest probability are atypical, the same reparametrization trick used throughout this paper to formalize this issue for continuous inputs is not directly applicable for discrete input spaces. However, similar adversarial constructions can be made in an analogous way: semantically close inputs can be considered distinct or identical depending on arbitrary choices of discretization/categorization [90], resulting in different probability values. Arbitrary choices of discretization include tokenization, lemmatization, or encoding see [91] for language modeling but also choice of language [92]. Figure 10 provides a similar construction in discrete pixel space.

Similarly, while approaches involving *probability masses* are unaffected by invertible reparametrizations, they explicitly rely on a deliberate choice in partitioning the input space, which is why we consider such approaches coherent with a more explicit incorporation of prior knowledge.

We make in the paper the assumption that the data distribution density $p_{X}^{*}$ is strictly positive everywhere in the set of possible instances X since in practice deep density models spread probability over all the input space. Arguably, an instance occurring outside the support of the data distribution would be considered an anomaly. An example would be CIFAR-10 and SVHN, which can be assumed to be disjoint. However, considering even the slightest Gaussian noise on either data distribution is sufficient to have *non-disjoint supports* as it makes the densities non-zero everywhere in the pixel space. Since Section 2.3 highlighted a failure of our geometrical intuition of density through the Gaussian Annulus theorem, we advocate for some skepticism on the assumption that these data distributions ought to be completely disjoint. In the general case, it is unknown whether anomalies lie outside of the distribution support and not uncommon to consider the probability of an anomaly happening to be non-zero with respect to the data distribution (i.e., $P_{X}^{*}\left(X_{out}\right)>0$), which is coherent with this strict positivity assumption. On the contrary, the concurrent work [84] chooses to assume a disjoint support for the inlier and outlier distributions, leading them to conclude that the model misestimation is the source of the observations made by Nalisnick et al. [27].

## 7. Broader Impact

Anomaly detection is commonly proposed as a fundamental element to safely deploy machine learning models in the real world. Its applications range from medical diagnostics and autonomous driving to cyber security and financial fraud detection.The use of such models on outlier points can result in dangerous behaviors but also discriminatory outcomes. Our paper aims at questioning current density-based anomaly detection methods, which is essential to mitigate the risks associated with their use in the real-world.

More broadly, our study also leads to reconsider the role of density as a standalone quantity and practices built around it, e.g., temperature sampling [21,44,45] and evaluating density models on anomaly detection, e.g., as in [34,93,94,95].

Finally, a common opinion in machine learning [96] has been that, given enough data and capacity, machine learning bias generally has a vanishing influence over the resulting bias in the learned solution. On the contrary, scale can obfuscate [82] misspecifications in the task and/or data collection design [97,98]. Here, we focused on how misspecifications in the algorithm design for anomaly detection can result in gross failure even in the ideal theoretical settings of infinite data and capacity.

However, this study provides a constructive proof in Section 4.2 that bad actors can use to arbitrarily manipulate the results of currently used anomaly detection algorithms, without modifying a learned model $p_{X}^{\left(\theta \right)}$. This opens the door to potential negative impacts if unreasonable trust in these methods are maintained in practice.

## Data Availability

Not applicable.

## Appendix A. Proof of Proposition 2

**Proposition** **A1.** *For any strictly positive density function $p_{X}^{*}$ over a convex space $X\subseteq R^{D}$ with D≥2, for any $x_{in},x_{out}$ in the interior $X^{o}$ of X, there exists a continuous bijection f:X→X such that $p_{X}^{*}=p_{f(X)}^{*}$, $p_{f(X)}^{*}\left(f\left(x^{\left(in\right)}\right)\right)=p_{X}^{*}\left(x^{\left(out\right)}\right)$, and $p_{f(X)}^{*}\left(f\left(x^{\left(out\right)}\right)\right)=p_{X}^{*}\left(x^{\left(in\right)}\right)$*.

**Proof.** Our proof will rely on the following non-rigid rotation $f^{\left(rot\right)}$. Working in a hyperspherical coordinate system consisting of a radial coordinate r>0 and (D−1) angular coordinates ${\left(\phi_{i}\right)}_{i<D}$,

$$\begin{array}{cc} \forall d<D,\;x_{d} & =r\left(\prod_{i=1}^{d-1}\sin(\phi_{i})\right)\cos\left(\phi_{d}\right)\\ x_{D} & =r\left(\prod_{i=1}^{D-2}\sin(\phi_{i})\right)\sin\left(\phi_{D-1}\right), \end{array}$$

where for all i∈{1,2,…,D−2},$\phi_{i}\in \left[0,\pi \right)$ and $\phi_{D-1}\in \left[0,2\pi \right)$, given $r_{max}>r_{0}>0$, we define the continuous mapping $f^{\left(rot\right)}$ as:

$$\begin{array}{cc} f^{\left(rot\right)}((r,\phi_{1}, & \cdots ,\phi_{D-2},\phi_{D-1}))\\ =(r,\phi_{1}, & \cdots ,\phi_{D-2},\\ \; & \phi_{D-1}+\pi \frac{{\left(r_{max}-r\right)}_{+}}{r_{max}-r_{0}}\left[\mathrm{mod}\;2\pi \right]). \end{array}$$

where ${\left(\cdot \right)}_{+}=\max\left(\cdot ,0\right)$. This mapping only affects points inside $B_{2}\left(0,r_{max}\right)$, and exchanges two points corresponding to $\left(r_{0},\phi_{1},\cdots ,\phi_{D-2},\phi_{D-1}\right)$ and $\left(r_{0},\phi_{1},\cdots ,\phi_{D-2},\phi_{D-1}+\pi \right)$ in a continous way (see Figure 7). Since the Jacobian determinant of the hyperspherical coordinates transformation is not a function of $\phi_{D-1}$, $f^{\left(rot\right)}$ is volume-preserving in cartesian coordinates. Let $f^{\left(KR\right)}$ be a Knothe-Rosenblatt rearrangement of $p_{X}^{*}$, $f^{\left(KR\right)}\left(X\right)$ is uniformly distributed in ${\left[0,1\right]}^{D}$. Let $z^{\left(in\right)}=f^{\left(KR\right)}\left(x^{\left(in\right)}\right)$ and $z^{\left(out\right)}=f^{\left(KR\right)}\left(x^{\left(out\right)}\right)$. Since $f^{\left(KR\right)}$ is continuous, $z^{\left(in\right)},z^{\left(out\right)}$ are in the interior ${\left(0,1\right)}^{D}$. Therefore, there is an ϵ>0 such that the $L_{2}$-balls $B_{2}\left(z^{\left(in\right)},\epsilon \right)$ and $B_{2}\left(z^{\left(out\right)},\epsilon \right)$ are inside ${\left(0,1\right)}^{D}$. Since ${\left(0,1\right)}^{D}$ is convex, so is their convex hull. Let $r_{0}=\frac{1}{2}{∥z^{\left(in\right)}-z^{\left(out\right)}∥}_{2}$ and $r_{max}=r_{0}+\epsilon$. Given $z\in {\left(0,1\right)}^{D}$, we write $z_{\|}$ and $z_{⊥}$ to denote its parallel and orthogonal components with respect to $\left(z^{\left(in\right)}-z^{\left(out\right)}\right)$. We consider the linear bijection *L* defined by

$$L\left(z\right)=z_{\|}+\epsilon^{-1}r_{max}z_{⊥}.$$

Let $f^{\left(z\right)}=L\circ f^{\left(KR\right)}$. Since *L* is a linear function (i.e., with constant Jacobian), $f^{\left(z\right)}\left(X\right)$ is uniformly distributed inside $L\left({\left[0,1\right]}^{D}\right)$. If $z^{\left(m\right)}$ is the mean of $z^{\left(in\right)}$ and $z^{\left(out\right)}$, then $f^{\left(z\right)}\left(X\right)$ contains $B_{2}\left(L\left(z^{\left(m\right)}\right),r_{max}\right)$ (see Figure 8). We can then apply the non-rigid rotation $f^{\left(rot\right)}$ defined earlier, centered on $L\left(z^{\left(m\right)}\right)$ to exchange $L\left(z^{\left(in\right)}\right)$ and $L\left(z^{\left(out\right)}\right)$ while maintaining this uniform distribution. We can then apply the bijection ${\left(f^{\left(z\right)}\right)}^{-1}$ to obtain the invertible map $f={\left(f^{\left(z\right)}\right)}^{-1}\circ f^{\left(rot\right)}\circ f^{\left(z\right)}$ such that $p_{f(X)}^{*}=f_{X}^{*}$, $p_{f(X)}^{*}\left(f\left(x^{\left(in\right)}\right)\right)=p_{X}^{*}\left(x^{\left(out\right)}\right)$, and $p_{f(X)}^{*}\left(f\left(x^{\left(out\right)}\right)\right)=p_{X}^{*}\left(x^{\left(in\right)}\right)$. □
